# Repeatability of Relative Free Energy Calculations in Solution with ANI-2x and MACE-OFF23

**DOI:** 10.1021/acs.jctc.5c01774

**Published:** 2025-12-17

**Authors:** Sara Tkaczyk, Thierry Langer, Marcus Wieder, Andrea Rizzi, Stefan Boresch

**Affiliations:** † Department of Pharmaceutical Sciences, Pharmaceutical Chemistry Division, University of Vienna, Josef-Holaubek-Platz 2, 1090 Vienna, Austria; ‡ Vienna Doctoral School of Pharmaceutical, Nutritional and Sport Sciences (PhaNuSpo), University of Vienna, 1090 Vienna, Austria; § Open Molecular Software Foundation, Davis, California 95616, United States; ∥ Computational Biomedicine, Institute of Advanced Simulations IAS-5/Institute for Neuroscience and Medicine INM-9, Forschungszentrum Jülich GmbH, 52428 Jülich, Germany; ⊥ Atomistic Simulations, 121451 Italian Institute of Technology, Genova 16163, Italy; # Faculty of Chemistry, Institute of Computational Biological Chemistry, University of Vienna, Währingerstrasse 17, 1090 Vienna, Austria

## Abstract

We investigate the feasibility and challenges of using neural network potentials (NNPs) for alchemical free energy calculations, employing a single-coordinate dual-topology approach. As a model application, we compute free energy differences between tautomer pairs to predict the preferred tautomeric state in aqueous solution. A central aspect of our approach is based on energy mixing via the selective masking of interactions involving dummy atoms, enabling a smooth interpolation between tautomeric states. This methodology is independent of the specific NNP architecture and holds potential for broader application to larger alchemical transformations. We tested this framework using two well-known NNPs: ANI-2x and MACE-OFF23­(small). While MACE-OFF23­(small) produced converged free energy results, simulations with ANI-2x showed significant variability across repeated runs. Our analysis traced this inconsistency to slow water dynamics and the overstabilization of artificial metastable states of the solute under ANI-2x, causing difficulties in converging sampling. Although transferable NNPs offer the advantage of general applicability without system-specific parametrization, our findings emphasize the importance of evaluating their performance in the condensed phase before employing them for free energy simulations.

## Graphical abstract

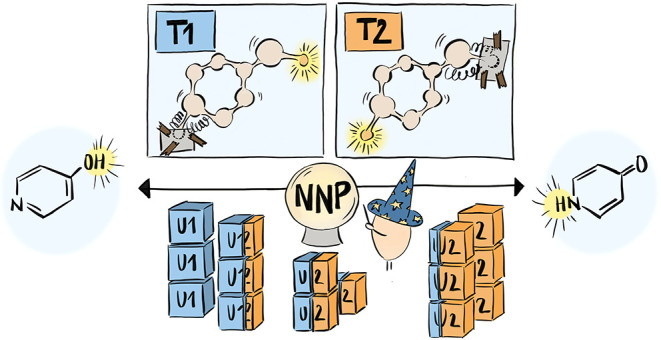

## Introduction

### Most Applications of Neural Network Potentials in Free Energy Calculations Involve NNP/MM Approaches

Free energy differences play a central role in governing biomolecular processes and chemical equilibria, but accurate modeling of molecular interactions in condensed phase systems remains a central challenge in computational chemistry.^1,2^ Classical molecular mechanics (MM) force fields (FF) have long been the default for molecular dynamics-based free energy calculations, largely because of their speed and well-established parametrization.^3,4^ However, their accuracy is still fundamentally limited, particularly when it comes to capturing the underlying quantum mechanical (QM) interactions. Recently, neural network potentials (NNPs) have emerged as a promising alternative and have shown strong predictive performance in reproducing quantum mechanical energies and forces at greatly reduced computational expense compared to *ab initio* methods.^5–7^ In the context of free energy calculations, NNPs have mostly been used in a hybrid fashion, typically by describing only a subsystem with the NNP.^8^ Examples using mechanical embedding include solvation^9,10^ and binding free energy studies.^11–14^ Electrostatic embedding schemes have also been explored.^15,16^ Another approach involves a custom-trained model that describes not only the internal energy of the solute but also its interactions with a surrounding shell of water molecules and was applied to the methanol to methane transformation.^17^ While these hybrid strategies retain the computational advantages of MM simulations, they also come with limitations, including challenges at the NNP–MM interface, the possible need for custom-trained interaction models, and artifacts arising from classical water models. These limitations motivate a move toward fully NNP-based simulations as an alternative path forward.

### Pretrained, Transferable NNPs Enable Fully NNP-Based Simulations

With recent advances in computational resources and the emergence of transferable, computationally efficient NNPs, first demonstrations of fully NNP-based simulations of entire solvated systems have become feasible, though they remain far from routine practice. Examples include simulations of organic molecules and small peptides in water, as well as exploratory studies of various solutes.^18–23^ This is noteworthy because of the nature of these potentials: The pretrained, transferable NNPs commonly used in computer-aided drug design and related fields are often trained on quantum chemical data for isolated molecules, dimers, and small water clusters.^24^ Combined with their relatively short interaction cutoffs,^5,25^ these models are highly effective in reproducing local, intramolecular interactions. Moreover, many model architectures were originally developed in the context of quantum chemistry and materials science, with an emphasis on accurate single-point energy prediction rather than for condensed phase behavior.^26,27^ In drug discovery, however, the goal is often to perform molecular dynamics simulations to obtain ensemble averages and free energy differences. Consequently, test set accuracy on energies and forces alone is insufficient to ensure robustness in condensed phase simulations.^28^ Furthermore, because NNP-based simulations are substantially more expensive than classical force field calculations, achieving adequate sampling remains a practical limitation, particularly for alchemical transformations where convergence is critical.

### Understanding Ensemble Effects of Pretrained NNPs is Essential for Free Energy Calculations

Understanding how pretrained, transferable NNPs capture ensemble properties is key for applications that rely on thermodynamic observables. Recent work has begun to explore this through studies of condensed phase properties of different solvents, including water.^29,30^ Within this context, two widely used transferable NNPs, ANI-2x^31^ and MACE-OFF23,^21^ have received considerable attention. Both models are readily available through platforms such as OpenMM-ML^32,33^ and are sufficiently efficient for full condensed phase NNP simulations. Notably, ANI-2x and MACE-OFF23 differ markedly in their reported ensemble properties for liquid water. Deviations were reported for properties such as radial distribution functions, diffusion coefficients, and density. For instance, ANI-2x has been reported to produce water that is overly structured, whereas MACE-OFF23 produces densities exceeding experimental measurements.^21,29,30,34^ Of all ensemble properties, free energy differences are among the most relevant for drug discovery. Recent fully NNP-based free energy studies have predominantly focused on absolute free energy calculations using “alchemy-aware” MACE-OFF23 models. These include applications in material design^35^ and absolute hydration free energies.^36^ Absolute hydration free energies for diverse organic molecules have also been reported using the Organic_MPNICE potential^37^ and the FeNNix-Bio1 ^38^ model, the latter additionally demonstrating a binding free energy calculation. We have also recently proposed an NNP-architecture-independent approach for computing absolute solvation free energies.^39^ In contrast, only limited work has been reported on relative free energies in full NNP frameworks and was restricted to nonperiodic systems.^40^

### Tautomers Are An Insightful Test Case for Alchemical, Relative Free Energy Calculations Using NNPs

In this work, we focus on the stability and convergence of relative free energy calculations in explicit solvent using two pretrained and widely used NNPs, ANI-2x and MACE-OFF23. We demonstrate how to perform such calculations robustly using an NNP combined with energy mixing (described in the Methods section below). We employ a single-coordinate dual-topology approach,^41,42^ technically realized by masking atoms. Since masked atoms do not interact with the rest of the system, restraints are applied to keep them from drifting away. In the context of QM/MM alchemical free energy simulations, such restraints have been referred to as “chaperoning potentials”. These are auxiliary MM restraints that keep end-point geometries meaningful when intramolecular interactions are removed by scaling the QM part of a QM/MM Hamiltonian to zero.^43^ As a model application, we set up a minimal alchemical transformation to investigate potential failure modes, specifically studying free energy differences between tautomer pairs to predict their preferred state in aqueous solution. This setup offers a tractable and meaningful test case: the alchemical transformation involves no changes in atom identity and only a shift in the position of a hydrogen atom, which can be implemented in an architecture-agnostic manner within an NNP framework. In addition, understanding tautomer preferences is highly relevant in drug discovery^44^ and accurate modeling of tautomer equilibria requires a level of theory beyond classical force fields, as full quantum mechanical approaches remain computationally prohibitive for routine use in condensed phase simulations.^45^ Apart from a single study on nonperiodic systems,^40^ relative free energies have not been investigated in fully periodic, solvated setups using pretrained NNPs, and the robustness of such calculations remains largely unexplored. Building on this test case of tautomer ratio calculations, this study revisits the problem under more realistic simulation conditions enabled by recent developments in methodology and computational resources. Key differences from previous work include the use of waterboxes with periodic boundary conditions to avoid vacuum exposure of solute molecules and artifacts from simulating in a water droplet environment. Simulation times were significantly extended from 100 ps to 1 ns per λ-state, and the time step increased to 1 fs. Importantly, we use the pretrained ANI-2x and MACE-OFF23 models without retraining on experimental data, and our goal is to assess methodological robustness rather than to reproduce experimental free energies, due to the different training objectives and data distributions of the two NNPs.

## Theory and Methods

### Single-Coordinate Dual-Topology Approach

The free energies in this work are calculated using the single-coordinate dual-topology approach.^41,42^ For tautomeric systems, this method employs a single coordinate set {*x⃗*}, representing the common substructure of both tautomers (highlighted in blue in Figure 1), along with two hydrogen atoms (highlighted in orange), which can either act as a dummy-atom (i.e., the noninteracting hydrogen in a given state) or a physical hydrogen atom in one of the tautomeric forms shown on the left and right sides in Figure 1. The energy of the physical endstates and the alchemical intermediate states *U*(*x⃗*; λ) is computed by mixing the energy of tautomer 1, *U* ^NNP^(*M*(*x⃗*, λ = 0)), and tautomer 2, *U* ^NNP^(*M*(*x⃗*, λ = 1)) and applying a set of restraints *U*_restr_^MM^(*x⃗*, λ) (described further down):

1 $$U\left(\mathrm{x⃗};\lambda \right)=\left(1-\lambda \right)U^{\mathrm{NNP}}\left(M\left(\mathrm{x⃗},\lambda =0\right)\right)+\lambda U^{\mathrm{NNP}}\left(M\left(\mathrm{x⃗},\lambda =1\right)\right)+U_{\mathrm{restr}}^{\mathrm{MM}}\left(\mathrm{x⃗},\lambda \right)$$

**Figure 1:**
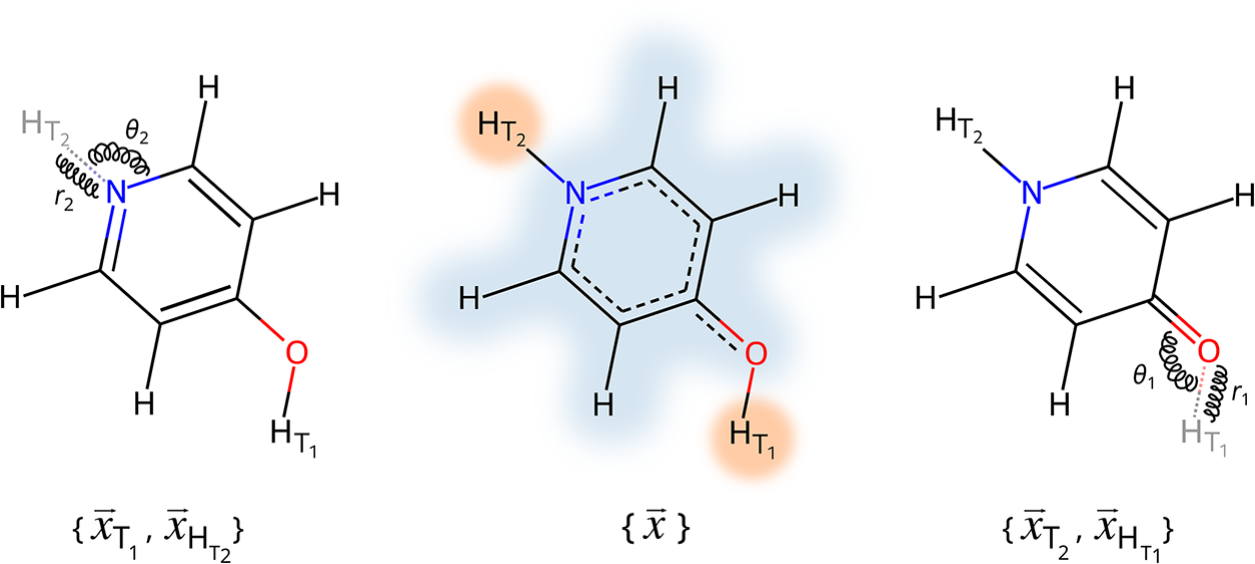
Visualization of the single-coordinate dual-topology approach. The molecular structure in the middle represents a set of coordinates that can be used to construct the topology of both tautomeric forms shown to the left and to the right, respectively. The subset of atoms common to both tautomeric forms is highlighted in blue; the hydrogen atoms that can act both as dummy and physical hydrogen atoms are highlighted in orange. In this example, the enol form *T*_1_ can be defined by masking *H*_T_2__ in the hybrid coordinate set {*x⃗*} and corresponds to λ = 0; the keto form can be described by masking *H*_T_1__, corresponding to λ = 1. Auxiliary restraints are shown as springs and are described in eq 2.

The correct topology for each tautomer is enforced by “masking” the dummy hydrogen in the hybrid coordinate set {*x⃗*}, ensuring it does not contribute to the energy evaluation. This is achieved with a masking function *M*(*x⃗*, λ). *M*(*x⃗*, λ = 0) masks the dummy atom *H*_T_2__, such that the NNP receives only the atomic coordinates corresponding to the topology of tautomer 1, denoted by {*x⃗*_T_1__} (i.e., the enol form in Figure 1). In turn, *M*(*x⃗*, λ = 1) masks dummy atom *H*_T_1__, yielding coordinates {*x⃗*_T_2__} of tautomer 2 (i.e., the keto form). The mixing is achieved with the coupling parameter λ, see eq 1. In practice, the masking function was implemented as follows: In the MACE-OFF23 ^21^ calculations, the dummy atom is masked by eliminating the respective atom index (and its corresponding neighbors/interactions) from the neighborlist before passing it to the neural network. In the torchani [Fn fn1] implementation of the ANI-2x potential^31,46^ this masking procedure can be achieved via a -1 flag in the species array. This approach has the same practical outcome as modifying the neighborlist directly. Thus, in principle, this masking strategy can be applied to any NNP that encodes atomic environments based on local neighborhoods within a specified cutoff.

Due to the masking procedure, special handling of the dummy atom is required, i.e., applying restraints inspired by those commonly employed in QM/MM free energy simulations to maintain structural integrity.^43^ The restraints used in this work constitute the *U*_restr_^MM^(*x⃗*, λ) term in eq 1 and are defined by the following functional form:

2 $$U_{\mathrm{restr}}^{\mathrm{MM}}\left(\mathrm{x⃗},\lambda \right)=U_{r}\left(\mathrm{x⃗},\lambda \right)+U_{\theta }\left(\mathrm{x⃗},\lambda \right)=\sum_{i=1}^{2}K_{r,i}\left(\lambda \right){\left(r_{i}-r_{0}\right)}^{2}+\sum_{i=1}^{2}K_{\theta ,i}\left(\lambda \right){\left(\theta_{i}-\theta_{0}\right)}^{2}$$

To prevent dissociation of the dummy atom from its neighboring heavy atom during the simulation, a λ-dependent harmonic bond restraint *U*_r_(*x⃗*, λ) is applied to the corresponding bond, as implemented in the HarmonicBondForce in OpenMM[Fn fn2].^32,33^ In Figure 1, this corresponds to applying a distance restraint between the oxygen and *H*_T_1__ (distance *r*_1_) to ensure that the dummy atom stays in vicinity of the oxygen. The restraint is active for all λ-states except the enol endstate itself, where *H*_T_1__ is a physical hydrogen. In turn, the same is true for the other dummy atom: a restraint is applied between the nitrogen atom and *H*_T_2__ (distance *r*_2_) in all λ-states except for the keto state. The harmonic bond restraints *U*_r_(*x⃗*, λ) are applied with an equilibrium distance *r*_0_ of 1 Å and λ-dependent force constants *K*_r,1_(λ) and *K*_r,2_(λ), associated with the distances *r*_1_ and *r*_2_, respectively. To improve phase-space overlap between consecutive λ-states, additional angle restraints are introduced. A λ-dependent harmonic angle restraint *U*_θ_(*x⃗*, λ) is applied to the angle between the dummy atom and two consecutive neighboring heavy atoms (HarmonicAngleForce). The corresponding angles are depicted in Figure 1 as θ_1_ and θ_2_. The angle restraints *U*_θ_(*x⃗*, λ) are applied with an equilibrium angle θ_0_ obtained from the minimized starting structure and with λ-dependent force constants *K*_θ,1_(λ) and *K*_θ,2_(λ). The λ-dependent, symmetric restraint scheme of the bond and angle force constants *K*_r,*i*_(λ) and *K*_θ,i_(λ) is shown in Table S1, using the bonds and angles depicted in Figure 1 as examples.

### System Setup

The hybrid structures required for the single-coordinate dual-topology approach were set up in the following way: first, the maximum common substructure (MCS) and the indices of both hydrogen atoms (that are not part of the MCS) were identified using the FindMSC function of the cheminformatics software package rdkit [Fn fn3].^47^ Next, the enol form of the tautomer was used to determine which heavy atom in the hybrid structure corresponds to the attachment point of the tautomeric hydrogen in the keto form. A dummy atom was attached to the identified heavy atom to create the hybrid structure. A 3D conformation was generated with rdkit. Reasonable coordinates of the dummy atom were found by metropolized Monte Carlo sampling. Candidate coordinates were randomly drawn from a sphere with fixed distance (1 Å) from the identified heavy atom and the energy was evaluated with ANI-2x (i.e., single point energies were computed with the ASE calculator [Fn fn4];^48^ subsequently, the structure was minimized with the BFGS algorithm). The minimized structure was solvated in a cubic 21 Å^3^ water box (≈290 water molecules) using the package PDBFixer [Fn fn5] available via OpenMM.

### Data Set

Reference values for all six tautomer pairs investigated in this work were taken from the Tautobase data set.^49,50^ Three of them were also part of the SAMPL2 challenge,^45^ a well-established benchmark set. The molecules were selected to be as small as possible with a minimal number of rotatable bonds to reduce potential sampling issues. The molecular structure of both tautomer forms can be seen in Figure 2. The SMILES strings, along with the corresponding reference free energy difference values, Δ*G*_ref_ are listed in Table S2. The reference values reported in the Tautobase were either determined experimentally or calculated via extrapolation using group contributions based on structurally related compounds. More details on the corresponding original sources are provided in Table S2.

**Figure 2:**
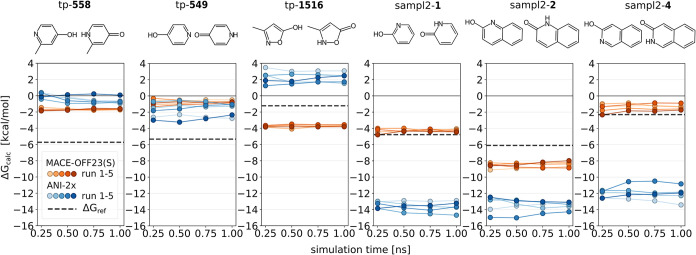
Computed free energy differences Δ*G*_calc_ compared to reference values Δ*G*_ref_ (dashed lines, see also Table S2) reported by Wahl and Sander,^49^ Taylor and Kenny^50^ and Geballe et al.^45^ for each tautomer pair. Results obtained with MACE are shown in shades of orange, while those from ANI are shown in shades of blue; five independent replicates are plotted for each potential. Free energy estimates were evaluated from increasing fractions of the trajectory (25%, 50%, 75%, and 100%) corresponding to 250 ps, 500 ps, 750 ps, and 1 ns of sampling per λ-state, respectively. The Δ*G*_calc_ values derived from the full 1 ns trajectories are listed in Table S4. Values of Δ*G*_calc_ below zero correspond to the keto form being preferred over the enol form.

### Free Energy Simulations

We aim to quantify the relative stability of tautomers in aqueous solution; i.e., we need to calculate the free energy difference between the two tautomers in the solvent phase. Note that in contrast to calculations of relative solvation free energy differences, no calculations in the gas phase are needed. The free energy difference between the two tautomeric forms was calculated with the staged free energy simulation method with 11 alchemical λ-states. The λ-scheme (mostly equidistant values between 0.0 and 1.0 with a step size 0.1, but additional intermediate states at 0.05 and 0.95 instead of 0.4 and 0.6) was chosen with a tighter spacing close to the endstates to ensure better phase-space overlap. Each λ-state was simulated for a total of 1.2 ns starting from a 21 Å cubic waterbox with periodic boundary conditions. OpenMM 8.2.0 was used in combination with the ANI-2x and MACE-OFF23­(S) NNPs available in the OpenMM-ML[Fn fn6] package. The torchani implementation was used for ANI-2x; the MACE-OFF23­(S) simulations were performed with the MACE-OFF23­(S) (small) model in float32. In both cases the energy evaluation needed to be adapted to account for the single-coordinate dual-topology scheme described above. In general, this approach requires passing the full set of coordinates (solute and solvent) through the network twice (except for λ = 0 and 1) in each simulation step, to account for both tautomer forms and allow for energy mixing described in eq 1. The initial velocities were drawn from a Maxwell–Boltzmann distribution at a temperature of 300 K. Coordinates were written every 1 ps, resulting in 1,200 samples per λ-state. A Langevin integrator was used with a time step of 1 fs and a collision rate of 1 ps. All simulations were performed in an NpT ensemble at a temperature of 300 K and a pressure of 1 atm, using the Monte Carlo Barostat available in OpenMM. Every simulation was repeated five times.

The first 200 samples (200 ps) were discarded for the subsequent free energy analysis to account for equilibration of the system. The tautomer free energy was calculated using every second sample, resulting in 11 × 500 (in total 5500) samples for the MBAR estimator,^51^ as implemented in the pymbar package[Fn fn7]. The code for running the described protocol can be found in the GitHub repository https://github.com/saratk1/tautomer_ratios.

### Practical Performance of Full-NNP Free Energy Calculations and Instabilities

On an NVIDIA RTX4090 GPU the present protocol reaches 4.7 ns day^–1^ with ANI and 2.2 ns day^–1^ with MACE-OFF23­(S) for the 900-atom systems studied here. During these simulations, we encountered occasional instabilities. The most frequent ones were solute deprotonation events involving the tautomeric hydrogens. To prevent such dissociation events, particularly near the endstates, a λ-independent flat-bottom harmonic bond restraint *U*_fb_(*x⃗*) was applied to both dummy atom–heavy atom bonds (*r*_1_ and *r*_2_ in Figure 1). This restraint remained constant in all λ-states with a force constant of 100 kcal/mol/Å^2^ and was activated at a distance *r*_fb_ of 1.5 Å (see eq 3):

3 $$U_{\mathrm{fb}}\left(\mathrm{x⃗}\right)=\sum_{i=1}^{2}1_{\left[r_{i}\geq r_{\mathrm{fb}}\right]}K_{\mathrm{fb}}{\left(r_{i}-r_{\mathrm{fb}}\right)}^{2}$$

The application of these restraints ensured that a consistent molecular topology was preserved during the simulations. Deprotonation events occurred primarily in float32 precision simulations, whereas test runs with float64 did not exhibit such behavior. However, the latter were significantly more computationally demanding and not feasible on our commodity hardware. A second source of instability involved sporadic water deprotonation events. In these cases, simulations were repeated starting with different random velocities. For MACE-OFF23­(S) simulations, crashes were first addressed by restarting the simulation, as trajectories could occasionally proceed without further issues. If instabilities persisted despite restarts, the time step was reduced from 1 to 0.5 fs to ensure stable propagation.

### Torsion Profiles and Potential Energy Decomposition

The torsion profiles shown in Figure 3 were extracted from the samples saved for the enol form of the corresponding tautomer at λ = 0. After discarding the first 200 ps, every frame was used to extract the C–C–O–H dihedral angle using mdtraj [Fn fn8].^52^

**Figure 3:**
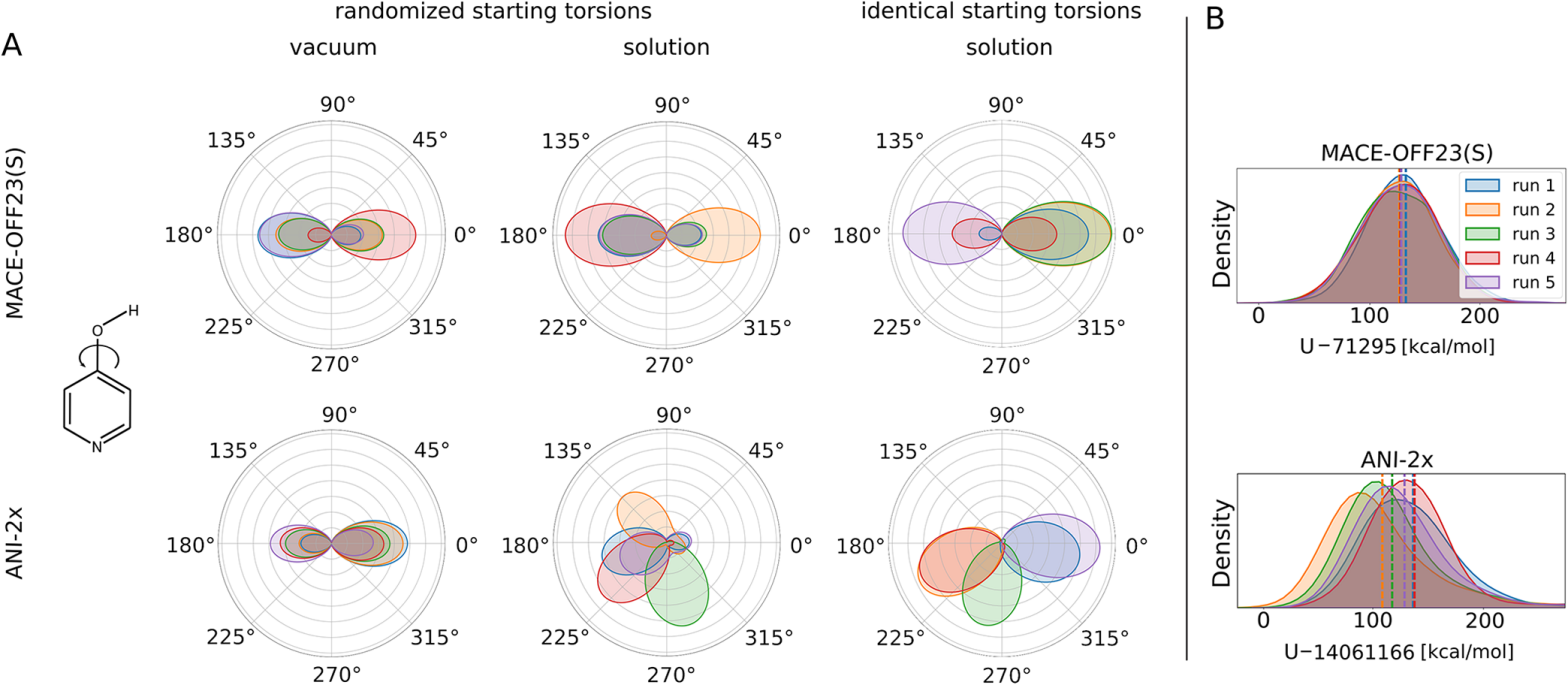
(A) Distributions of the C–C–O–H dihedral in tautomer tp-**549** at λ = 0 (enol form). MACE results are shown in the top row and ANI results in the bottom row under three conditions: gas phase with randomized initial torsions (first column), aqueous solution with randomized initial torsions (second column), and aqueous solution with identical initial torsions (third column). Five independent repeats are color-coded within each panel. (B) Corresponding potential energy distributions for the solvated system (tp-**549**) at λ = 0. Five repeats are shown for MACE and for ANI. Energies are plotted relative to the minimum value observed for the respective potential (*U*_min_ = 71295 kcal mol^–1^ and *U*_min_ = 14061166 kcal mol^–1^ for MACE and ANI, respectively), yielding a zero offset. The *x*-axis range is identical for ANI and MACE. Dashed vertical lines mark the average potential energy of each repeat.

For the potential energy decomposition, the system was split into solute and solvent components (*U*_solute–solute_ and *U*_solvent–solvent_, respectively), and the potential energies were reevaluated with the corresponding NNP for all saved frames using OpenMM. The solute–solvent interaction energy *U*_solute–solvent_ was then calculated as *U*_solute–solvent_ = *U*_total_ – (*U*_solvent–solvent_ + *U*_solute–solute_), with *U*_total_ as the total potential energy of the system.

## Results and Discussion

### Foundation Models Trained on QM Data Can Exhibit Different Convergence of Alchemical Free Energy Calculations in Solution

To assess how different pretrained NNPs affect the feasibility of alchemical free energy calculations in solution, we compared the convergence behavior of ANI-2x and MACE-OFF23­(S) for predicting the relative stability of tautomer pairs in explicit water, using six representative pairs from the Tautobase and SAMPL2 data sets. For brevity, these models are referred to as ANI and MACE in the following. Convergence and reproducibility were evaluated by performing five independent repeats of each calculation starting from the same initial structure, and the results are summarized in Figure 2. Overall, MACE showed substantially better agreement across repeats than ANI, and its consistency improved with longer simulations. In contrast, ANI displayed no clear trend with simulation length. Across all systems, the standard deviation of the free energy estimates σ­(Δ*G*) was consistently lower for MACE (0.1–0.3 kcal mol^–1^) than for ANI (0.4–0.8 kcal mol^–1^) (for details, see Table S3). The spread between the minimum and maximum ANI free energy values ΔΔ*G*_min,max_ ranged from 1.0 to 2.6 kcal mol^–1^, while the corresponding range for MACE remained below 0.9 kcal mol^–1^ (see Table S3). While no clear trend was observed for ANI, MACE showed increased standard deviations for the larger systems (i.e., sampl2-**2** and sampl2-**4**). Despite attempts to optimize the protocol, ANI exhibited sizable deviations between independent runs, in contrast to MACE where agreement between repeats was in line with expectation.

The free energy differences reported above are shown relative to literature reference values Δ*G*_ref_ compiled by Wahl and Sander,^49^ Taylor and Kenny^50^ and Geballe et al.^45^ (see Table S2 for more details). Across the six systems, ANI exhibits a root-mean-square error (RMSE) of 6.84 [5.91;7.69] kcal mol^–1^ and a mean absolute error (MAE) of 6.36 [5.41;7.33] kcal mol^–1^, whereas MACE reduces these values to 2.90 [2.38;3.38] and 2.49 [1.92;3.04] kcal mol^–1^, respectively (95% confidence intervals from 1000 bootstrap replicates with replacement).

We recently studied how well several NNPs, including ANI-2x and MACE-OFF­(S) used here, reproduce condensed phase properties of water and five organic liquids,^30^ and found that none of the models performed well. The mediocre agreement with experiment is not surprising, not only due to the training objectives (formation energies) and the training data set, but also given that the NNPs used in this study and our earlier work^30,39^ were trained neither on condensed phase properties nor on solvation free energies. Despite these limitations, comparison with reference data shows that MACE led to converged results across multiple repeats and was in better agreement with experiments than ANI. Moreover, MACE consistently predicted the correct tautomer preference for all systems, whereas ANI reversed the preference in one tautomer pair across all five repeats (tp-**1516**) and failed in a single repeat for another pair (tp-**558**, see also Figure 2 and Table S4).

### Slow and Heterogeneous Water Dynamics Explain the Poor Performance of ANI

Convergence problems in alchemical free energy calculations can arise from inadequate sampling of key regions, such as the conformational substates of the solute. To investigate this, we thus first analyzed solute behavior in our test set. In particular, we focused on the hydroxy groups of the enol forms of the tautomers, which might be prone to challenging torsional flexibility. In vacuum, torsion distributions of the C–C–O–H torsion angle of the solute showed excellent agreement across independent runs for both ANI and MACE, indicating that the solute alone is not responsible for the observed discrepancies. An example of the C–C–O–H dihedral distributions of tp-**549** (corresponding to five simulations at λ = 0) is shown in panel A of Figure 3. When simulations were performed in explicit water, marked differences emerged between the two NNPs: In the shown example, MACE samples similar C–C–O–H torsion distributions in vacuum and in solution. By contrast, however, ANI produces widely different angles between vacuum and solvent and also shows variability across independent runs, even when starting from the same conformation (see Figure 3, panel A). Similar behavior was observed for the other tautomer pairs at λ = 0, as well as at intermediate λ-states close to the enol end state (i.e., states up to λ = 0.5).

To further probe the origin of the convergence issues, we examined ensemble-averaged quantities such as the total potential energy of the system. For ANI, deviations in the mean *U* _tot_ of up to 30 kcal mol^–1^ were observed between nominally identical repeats, whereas MACE showed consistent mean potential energies across runs (for details, see Tables S5 and S6). The potential energy distributions of the enol simulations (i.e., at λ = 0) of the example molecule tp-**549** are shown in panel B of Figure 3. Despite the inconsistent solute torsion profiles produced by ANI (panel A of Figure 3), it is unlikely that solute conformational differences alone account for the large deviations of up to 30 kcal mol^–1^. To confirm the source of these variations, we performed a potential energy decomposition, examining contributions from the solute, solvent, and solute–solvent interactions separately. Indeed, the average solute–solute energies were very similar and inconspicuous across repeats, even though the dihedral angle distribution varied markedly, reinforcing that differences in free energy convergence likely stem from the solvent environment. Across independent runs of the same system, the mean water–water interaction energy *U* _solvent–solvent_^min, max^ varied by up to 34 kcal mol^–1^ for ANI, compared to only 8 kcal mol^–1^ for MACE (for details, see Table S7). This problem was not limited to a single λ-state; similar variations were observed across the entire alchemical range (see Figure S1 for an example at λ = 0 and λ = 1). We observed analogous discrepancies in potential energy distributions for pure-water boxes (see Figure S1), pointing to a fundamental solvent issue. This indicates that slow and heterogeneous solvent dynamics, rather than solute–solute or solute–solvent interactions alone, drive poor reproducibility.

Such behavior is further reflected in the prolonged water–solute residence times observed for ANI, where individual waters remain hydrogen-bonded to the solute hydroxyl-group for up to ≈2 ns, compared with picosecond time scales for MACE (see Figure S2). These findings complement earlier reports of overly structured radial distribution functions for ANI water and significantly lower water diffusion (approximately 3 orders of magnitude lower than the experimental value).^21,29,30^

Overall, the incorrect solvent dynamics in ANI prevent both solvent and solute from equilibrating properly, resulting in sampling of distinct conformational spaces and unconverged free energy estimates. In contrast, MACE reproduces realistic solvent dynamics, allowing equilibration and yielding converged results across independent runs. These observations underline that, even for a minimal alchemical transformation, the choice of NNP critically determines whether reliable convergence can be reached within practical simulation times. Routine validation against basic solvent metrics such as diffusion coefficients, residence times, and potential energy distributions is therefore essential before performing production free energy calculations.

## Conclusion

This work set out to evaluate whether modern, transferable NNPs can deliver robust alchemical free energy calculations in explicit solvent without system-specific reparameterization. Using a single-coordinate dual-topology protocol for six tautomer pairs, we compared two widely adopted models, ANI-2x and MACE-OFF23­(S), under identical simulation conditions. Only MACE-OFF23­(S) achieved reproducible convergence within the practical limit of 1 ns per λ-state, yielding free energy estimates whose standard deviation across five independent repeats remained below 0.3 kcal mol^–1^ and whose root-mean-square error against experimental reference data was roughly 3 kcal mol^–1^. By contrast, ANI-2x exhibited standard deviations up to 0.8 kcal mol^–1^, average deviations from reference values around 6 kcal mol^–1^, and large run-to-run fluctuations that failed to diminish with additional sampling.

Detailed diagnostics traced ANI-2x’s poor behavior to heterogeneous water dynamics: solvent–solvent interaction energies varied by tens of kilocalories per mole between repeats, individual water molecules remained in close contact with hydroxyl groups of the solute for nanoseconds, and the resulting torsional trapping of the solute prevented sufficient exploration of the phase space. These artifacts, absent in MACE-OFF23­(S) and already hinted at by ANI-2x’s radial distribution function and diffusion coefficient anomalies reported elsewhere, underscore that condensed phase validation must precede any production use of an NNP in free energy workflows. In fact, the severe slowdown of solvent dynamics effectively violates the ergodic assumption underlying the estimation of ensemble averages from molecular dynamics simulations, making it impossible to obtain converged results within simulation times that are typically sufficient in MM simulations.

From a practical standpoint, full-system alchemical simulations with NNPs are now feasible on commodity GPUs—our protocol reached ≈4.7 ns day^–1^ with ANI-2x and ≈2.2 ns day^–1^ with MACE-OFF23­(S) for 900 atom boxes. Nonetheless, these simulations are still orders of mangnitude more expensive than classical force field calculations. Occasional instabilities can require stricter numerical settings, such as reducing the time step to 0.5 fs or switching to double precision, to prevent crashes or deprotonations. Although MACE-OFF23­(S) generally achieves better convergence, it can also produce unstable trajectories, highlighting that stability remains a practical limitation.

Unlike traditional force fields, which embed domain-specific assumptions into the potential energy function, NNPs rely entirely on data-driven learning. This makes not only the model architecture but also the molecular representation and, critically, the training set design central to achieving transferability across chemical space. Our findings suggest that while MACE-OFF23­(S) represents meaningful progress, robust condensed phase performance will ultimately depend on training sets that better capture intermolecular interactions and solvent behavior.

## Supplementary Material



## Data Availability

Python package used in this work: https://github.com/saratk1/tautomer_ratios.

## Footnotes

a
https://github.com/aiqm/torchani.

b
https://github.com/openmm.

c RDKit: Open-source cheminformatics https://www.rdkit.org.

d
https://github.com/DeepChoudhuri/Atomic-Simulation-Environment.

e
https://github.com/openmm/pdbfixer.

f
https://github.com/openmm-ml.

g
https://github.com/choderalab/pymbar.

h
https://github.com/mdtraj/mdtraj.

## Notes

Table S1 lists the force constants *K*_r,*i*_ and *K*_θ,i_ used in the harmonic bond and angle restraints. Table S2 provides SMILES strings for all tautomer pairs included in this study along with their corresponding reference free energy difference values and the original sources. Table S3 reports the standard deviation σ­(ΔG) of the free energy difference from five runs, as well as the range between the lowest and highest calculated free energy differences ΔΔ*G*_min,max_ (for ANI-2x and MACE-OFF23­(S)). Table S4 summarizes the calculated free energy differences from five repeats and the associated deviations from the reference values for both potentials. Tables S5 and S6 present the mean of the potential energy at λ = 0 obtained from five simulations for each tautomer system, for ANI-2x and MACE-OFF23­(S), respectively. Further details on the potential energy decomposition are also included. Table S7 reports the range of minimum and maximum mean potential energies across five runs for both NNPs. Figure S1 illustrates the potential energy distributions of water molecules extracted from tp-**549** solute–solvent simulations at λ = 0, λ = 1, and from pure water box simulations without solute. Finally, Figure S2 presents the residence time analysis of water molecules near the OH group of one of the tautomers in the enol form (PDF)
