# Supplementary material for: Repeatability of Relative Free Energy Calculations in Solution with ANI-2x and MACE-OFF23
Source: J Chem Theory Comput. 2025 Dec 17;22(1):231–9. doi: 10.1021/acs.jctc.5c01774 (PMC12805565; doi:10.1021/acs.jctc.5c01774)
Supplement: Supplementary file 1 [file ct5c01774_si_001.pdf]

Supplementary Infomation: Repeatability of Relative Free Energy Calculations in Solution with ANI-2x and MACE-OFF23

Sara Tkaczyk,[†,‡] Thierry Langer,[†] Marcus Wieder,[¶] Andrea Rizzi,[*,§,‖] and Stefan Boresch[*,⊥]

†*Department of Pharmaceutical Sciences, Pharmaceutical Chemistry Division, Josef-Holaubek-Platz 2, University of Vienna, 1090 Vienna, Austria*

‡*Vienna Doctoral School of Pharmaceutical, Nutritional and Sport Sciences (PhaNuSpo), University of Vienna, Vienna, Austria*

¶*Open Molecular Software Foundation, Davis, California 95616, United States*

§*Computational Biomedicine, Institute of Advanced Simulations IAS-5/Institute for Neuroscience and Medicine INM-9, Forschungszentrum Jülich GmbH, Jülich 52428, Germany*

‖*Atomistic Simulations, Italian Institute of Technology, Genova 16163, Italy*

⊥*Faculty of Chemistry, Institute of Computational Biological Chemistry, Währingerstrasse 17, University of Vienna, Vienna, Austria*

E-mail: a.rizzi@extern.fz-juelich.de; stefan@mdy.univie.ac.at

Table S1: Force constants $K_{\mathrm{r,i}}$ and $K_{\theta\mathrm{,i}}$ used in the harmonic bond and angle restraints listed for all λ-states, following the nomenclature in Figure 1 of the main manuscript. At the physical-endstates, where H_{T_1} or H_{T_2} are physical hydrogen atoms, no restraints are applied ($K_{\mathrm{r,1}} = 0$ and $K_{\mathrm{r,2}} = 0$). The restraint $K_{\mathrm{r,1}}$ is turned on, starting with a low force constant of 1 kcal mol^{-1} Å^{-2} at $\lambda = 0.05$ and increases linearly until $K_{\mathrm{r,1}} = 100$ kcal mol^{-1} Å^{-2}, according to $K_{\mathrm{r,1}}(\lambda) = 1 + \frac{99}{0.95}(\lambda - 0.05),\ 0.05 \leq \lambda \leq 1$. $K_{\mathrm{r,2}}$ is turned off linearly, following $K_{\mathrm{r,2}}(\lambda) = 100 - \frac{99}{0.95}\lambda,\ 0 \leq \lambda \leq 0.95$ until it reaches $K_{\mathrm{r,2}} = 1$ kcal mol^{-1} Å^{-2} at $\lambda = 0.95$. The same scheme is applied to angle restraints, where the values of the force constants $K_{\theta\mathrm{,1}}$ and $K_{\theta\mathrm{,2}}$ are given in kcal mol^{-1} rad^{-2}.

λ	0.00	0.05	0.10	0.20	0.30	0.50	0.70	0.80	0.90	0.95	1.00
$K_{\mathrm{r,1}}$ [kcal mol^{-1} Å^{-2}]	0.00	1.00	6.21	16.6	27.1	47.9	68.7	79.2	89.6	94.8	100
$K_{\mathrm{r,2}}$ [kcal mol^{-1} Å^{-2}]	100	94.8	89.6	79.2	68.8	47.9	27.1	16.6	6.21	1.00	0.00
$K_{\theta\mathrm{,1}}$ [kcal mol^{-1} rad^{-2}]	0.00	0.10	0.62	1.66	2.71	4.79	6.87	7.92	8.96	9.48	10.0
$K_{\theta\mathrm{,2}}$ [kcal mol^{-1} rad^{-2}]	10.0	9.48	8.96	7.92	6.87	4.79	2.71	1.66	0.62	0.10	0.00

Table S2: SMILES strings of the tautomer pairs studied in this work, along with the original source and the corresponding reference ΔG_{ref} value. Tautomer preferences for all six tautomer pairs are available in the Tautobase;[1,2] three pairs were also part of the SAMPL2 challenge[3] (the corresponding Tautobase numbering is given as a tablenote). Negative ΔG_{ref} values indicate preference for the keto form (tautomer 2) over the enol form (tautomer 1). We provide the error estimates in parentheses for the three pairs from the SAMPL2 challenge; see Table 2 of Ref. 3. Note that the SAMPL2 publication[3] reports experimental ΔG_{ref} values and error estimates without citation of experimental sources. Where available, we list the reference for the original experimental work, as cited in Tautobase.[1,2] For two tautomer pairs (tp-**558** and tp-**1516**), the Tautobase only provides extrapolated log K values from group contribution based on structurally related compounds. In these cases, a tablenote points to the table in Ref. 1 we used. The log K values available in the Tautobase were converted to the free energy difference ΔG_{ref} by using $\Delta G = -2.303 \cdot R \cdot T \cdot \log K$, with $R = 1.987 \times 10^{-3}$ kcal $\cdot$ mol$^{-1} \cdot$ K^{-1} and $T = 298$ K.

tautomer pair	SMILES tautomer 1	SMILES tautomer 2	ΔG_{ref} [kcal mol^{-1}]
tp-**558**[1] *	`Cc1ncccOc1`	`CCNC=C1=CC1=O`	-5.7
tp-**549**[4]	`Oc1ccncc1`	`O=C1C=CNC=C1`	-5.3
tp-**1516**[1] †	`Cc1nocOc1`	`CCNO1=CC1=O`	-1.2
sampl2-**1**[5] ‡	`Oc1ncccc1`	`O=C1NC=CC=C1`	-4.8 (0.3)
sampl2-**2**[5] §	`OC1=CC=C2C=CC=CC2=N1`	`O=C1NC2=CC=CC=C2C=C1`	-6.1 (0.3)
sampl2-**4**[6] ¶	`OC1=CC2=CC=CC=C2C=N1`	`O=C1NC=C2C=CC=CC2=C1`	-2.3 (0.4)

*Table 37 in Ref. 1

†Table 83 in Ref. 1

‡also in Tautobase: tp-**982**

§also in Tautobase: tp-**997**

¶also in Tautobase: tp-**101**

Table S3: Standard deviation $\sigma(\Delta G)$ of the free energy difference from five runs obtained with MACE-OFF23(S) and ANI-2x. $\Delta\Delta G_{\text{min,max}}$ denotes the range between the lowest and highest calculated ΔG. All free energy results are given in kcal mol^{-1}.

tautomer pair	**MACE-OFF23(S)** $\sigma(\Delta G)$ [kcal mol^{-1}]	**MACE-OFF23(S)** $\Delta\Delta G_{\text{min,max}}$ [kcal mol^{-1}]	**ANI-2x** $\sigma(\Delta G)$ [kcal mol^{-1}]	**ANI-2x** $\Delta\Delta G_{\text{min,max}}$ [kcal mol^{-1}]
tp-**558**	0.1	0.2	0.4	1.0
tp-**549**	0.1	0.4	0.7	1.7
tp**1516**	0.1	0.3	0.6	1.5
sampl2-**1**	0.2	0.4	0.6	1.8
sampl2-**2**	0.3	0.9	0.4	1.2
sampl2-**4**	0.3	0.8	0.8	2.6

Table S4: Free energy differences ΔG_r for each of the five runs ($r_1 - r_5$) of the same protocol for MACE-OFF23(S) and ANI-2x. The deviation to the reference (ref) free energy value is calculated as $\delta\Delta G_{\text{ri,ref}} = \Delta G_{\text{ri}} - \Delta G_{\text{ref}}$. The reference value can be found in Table S2. All free energy results are given in kcal mol^{-1}.

tautomer pair	ΔG_{r_1}	ΔG_{r_2}	ΔG_{r_3}	ΔG_{r_4}	ΔG_{r_5}	$\delta\Delta G_{r_1,\text{ref}}$	$\delta\Delta G_{\text{r2,ref}}$	$\delta\Delta G_{\text{r3,ref}}$	$\delta\Delta G_{\text{r4,ref}}$	$\delta\Delta G_{\text{r5,ref}}$
**MACE-OFF23(S)**										
tp-**558**	-1.58	-1.74	-1.67	-1.63	-1.59	4.15	3.99	4.06	4.10	4.14
tp-**549**	-0.49	-0.71	-0.79	-0.85	-0.74	4.83	4.61	4.53	4.47	4.58
tp-**1516**	-3.69	-3.68	-3.70	-3.56	-3.81	-2.46	-2.45	-2.47	-2.00	-2.58
sampl2-**1**	-4.05	-4.17	-4.26	-4.47	-4.43	0.73	0.61	0.52	0.31	0.35
sampl2-**2**	-8.68	-8.21	-8.37	-8.87	-7.98	-2.58	-2.11	-2.27	-2.77	-1.88
sampl2-**4**	-1.30	-1.34	-1.61	-0.89	-1.74	1.00	0.96	0.69	1.41	0.56
**ANI-2x**										
tp-**558**	-0.64	-0.95	-0.8	-0.73	0.08	5.09	4.78	4.93	5.00	5.81
tp-**549**	-2.76	-1.26	-1.12	-1.06	-2.34	2.56	4.06	4.20	4.26	2.98
tp-**1516**	3.07	1.53	2.48	1.71	2.46	4.30	2.76	3.71	2.94	3.69
sampl2-**1**	-12.89	-13.72	-14.69	-13.65	-13.22	-8.11	-8.94	-9.91	-8.87	-8.44
sampl2-**2**	-13.11	-13.61	-13.37	-14.26	-13.09	-7.01	-7.51	-7.27	-8.16	-6.99
sampl2-**4**	-13.42	-12.28	-11.85	-10.83	-11.95	-11.12	-9.98	-9.55	-8.53	-9.65

Table S5: Mean of the potential energy at $\lambda = 0$ from five MACE-OFF23(S) simulations for each tautomer system, shown for the total system $\overline{U}_{\text{total}}$, solute $\overline{U}_{\text{solute-solute}}$, solvent $\overline{U}_{\text{solvent-solvent}}$, and solute–solvent contributions $\overline{U}_{\text{solute-solute}}$. The 95% confidence intervals were estimated from 1,000 bootstrap replicates with replacement. The potential energy values are reported relative to the system-specific minimum; the original absolute potential energy can be recovered by adding the offset to the reported values.

taut. pair	run or offset	$\overline{U}_{\text{total}}$	$\overline{U}_{\text{solute-solute}}$	$\overline{U}_{\text{solvent-solvent}}$	$\overline{U}_{\text{solute-solvent}}$
		**MACE-OFF23(S)**			
tp-**558**	offset	-71345.16	-1638.65	-69663.39	-66.83
	run 1	124.82 [122.48, 127.45]	8.52 [8.36, 8.67]	119.34 [116.99, 121.91]	20.72 [20.42, 21.01]
	run 2	122.96 [120.65, 125.61]	8.57 [8.42, 8.74]	117.29 [115.00, 119.88]	20.82 [20.51, 21.14]
	run 3	129.01 [126.65, 131.44]	8.72 [8.55, 8.88]	123.26 [120.89, 125.74]	20.76 [20.45, 21.04]
	run 4	123.12 [120.90, 125.49]	8.67 [8.50, 8.84]	117.14 [114.83, 119.77]	20.97 [20.64, 21.26]
	run 5	129.19 [126.81, 131.62]	8.61 [8.45, 8.77]	123.48 [121.22, 126.05]	20.75 [20.43, 21.05]
tp-**549**	offset	-71295.38	-1340.31	-69920.43	-61.67
	run 1	134.59 [132.17, 137.21]	6.60 [6.45, 6.74]	135.89 [133.70, 138.64]	19.05 [18.77, 19.34]
	run 2	128.61 [126.23, 131.15]	6.56 [6.40, 6.72]	130.33 [128.01, 132.92]	18.70 [18.43, 18.97]
	run 3	129.51 [127.19, 131.98]	6.67 [6.52, 6.82]	130.89 [128.45, 133.34]	18.98 [18.69, 19.29]
	run 4	129.55 [127.24, 132.04]	6.60 [6.45, 6.76]	131.03 [128.76, 133.67]	18.92 [18.63, 19.20]
	run 5	130.37 [127.82, 132.88]	6.56 [6.41, 6.71]	131.99 [129.56, 134.71]	18.81 [18.53, 19.10]
tp-**1516**	offset	-70945.64	-1250.30	-69660.17	-126.08
	run 1	118.46 [116.08, 121.05]	10.42 [9.70, 11.16]	116.22 [113.92, 118.68]	82.70 [81.71, 83.66]
	run 2	113.92 [111.32, 116.37]	7.51 [7.35, 7.68]	111.53 [109.08, 113.98]	85.69 [85.36, 86.02]
	run 3	116.90 [114.58, 119.32]	7.26 [7.10, 7.42]	114.20 [111.87, 116.59]	86.48 [86.16, 86.82]
	run 4	116.90 [114.61, 119.55]	7.63 [7.46, 7.78]	115.29 [113.08, 117.74]	84.89 [84.58, 85.21]
	run 5	114.35 [112.21, 116.65]	7.65 [7.48, 7.81]	112.38 [110.02, 114.71]	85.27 [84.97, 85.57]
sampl2-**1**	offset	-71756.72	-1348.39	-70379.68	-72.99
	run 1	108.26 [105.81, 110.73]	7.85 [7.69, 8.00]	106.98 [104.61, 109.53]	37.76 [37.48, 38.05]
	run 2	106.25 [103.71, 108.78]	8.49 [8.31, 8.65]	105.87 [103.34, 108.38]	36.26 [35.90, 36.58]
	run 3	105.65 [103.28, 108.24]	9.43 [9.23, 9.61]	106.49 [104.04, 108.93]	33.98 [33.56, 34.36]
	run 4	105.18 [102.87, 107.70]	7.83 [7.68, 7.98]	103.85 [101.67, 106.33]	37.84 [37.55, 38.10]
	run 5	108.59 [106.19, 111.20]	10.86 [10.69, 11.04]	111.68 [109.14, 114.08]	30.39 [30.00, 30.78]
sampl2-**2**	offset	-71832.46	-2138.69	-69657.09	-78.32
	run 1	114.94 [112.73, 117.48]	9.89 [9.68, 10.11]	115.22 [112.88, 117.80]	31.56 [31.19, 31.91]
	run 2	115.48 [113.00, 117.98]	10.59 [10.38, 10.79]	116.95 [114.55, 119.40]	29.61 [29.18, 30.05]
	run 3	113.97 [111.63, 116.38]	12.82 [12.63, 13.02]	119.02 [116.78, 121.37]	23.75 [23.39, 24.09]
	run 4	115.79 [113.25, 118.56]	9.68 [9.48, 9.89]	115.75 [113.13, 118.36]	32.01 [31.64, 32.45]
	run 5	112.67 [110.25, 115.34]	11.94 [11.74, 12.15]	116.38 [113.81, 118.90]	25.98 [25.58, 26.41]
sampl2-**4**	offset	-72077.91	-2135.95	-69909.25	-74.53
	run 1	121.36 [118.84, 123.95]	12.15 [11.96, 12.34]	128.44 [125.97, 130.91]	22.60 [22.21, 22.98]
	run 2	122.58 [120.29, 125.32]	11.43 [11.24, 11.65]	127.85 [125.52, 130.34]	25.19 [24.79, 25.62]
	run 3	122.06 [119.68, 124.68]	11.40 [11.21, 11.59]	127.43 [125.14, 130.01]	25.08 [24.67, 25.49]
	run 4	119.10 [116.58, 121.75]	10.92 [10.73, 11.13]	123.67 [121.19, 126.18]	26.39 [25.96, 26.79]
	run 5	121.63 [119.23, 124.04]	9.88 [9.69, 10.07]	124.18 [121.80, 126.63]	29.46 [29.12, 29.82]

Table S6: Mean of the potential energy at $\lambda = 0$ from five ANI-2x simulations for each tautomer system, shown for the total system $\overline{U}_{\text{total}}$, solute $\overline{U}_{\text{solute-solute}}$, solvent $\overline{U}_{\text{solvent-solvent}}$, and solute–solvent contributions $\overline{U}_{\text{solute-solute}}$. The 95% confidence intervals were estimated from 1,000 bootstrap replicates with replacement. The potential energy values are reported relative to the system-specific minimum; the original absolute potential energy can be recovered by adding the offset to the reported values.

taut. pair	run or offset	$\overline{U}_{\text{total}}$	$\overline{U}_{\text{solute-solute}}$	$\overline{U}_{\text{solvent-solvent}}$	$\overline{U}_{\text{solute-solvent}}$
		**ANI-2x**			
tp-**558**	offset	-14037861.85	-227610.29	-13810225.57	-59.63
	run 1	117.92 [114.88, 121.47]	8.24 [8.06, 8.42]	113.85 [110.66, 117.45]	29.54 [29.06, 29.99]
	run 2	117.56 [114.15, 121.40]	7.86 [7.69, 8.04]	117.99 [114.69, 121.65]	25.35 [24.92, 25.85]
	run 3	90.14 [86.47, 94.06]	8.76 [8.58, 8.93]	96.79 [93.21, 100.74]	18.21 [17.91, 18.49]
	run 4	120.44 [117.12, 124.10]	9.42 [9.24, 9.60]	122.72 [119.34, 126.95]	22.00 [21.61, 22.37]
	run 5	117.37 [114.12, 121.42]	9.07 [8.88, 9.25]	118.97 [115.71, 122.95]	22.91 [22.58, 23.22]
tp-**549**	offset	-14061166.03	-202943.42	-13858187.70	-59.75
	run 1	136.85 [133.36, 140.47]	6.95 [6.79, 7.11]	122.34 [119.14, 126.21]	32.41 [32.12, 32.67]
	run 2	108.66 [105.15, 112.46]	7.60 [7.44, 7.75]	101.59 [98.08, 105.39]	24.26 [23.90, 24.67]
	run 3	117.62 [114.34, 121.34]	9.61 [9.41, 9.79]	102.89 [99.44, 106.43]	29.98 [29.67, 30.29]
	run 4	137.98 [134.93, 141.64]	7.51 [7.34, 7.68]	132.48 [129.29, 136.26]	22.94 [22.67, 23.22]
	run 5	129.04 [125.69, 132.98]	6.80 [6.65, 6.96]	123.35 [120.23, 126.86]	23.64 [23.30, 23.99]
tp-**1516**	offset	-14036486.06	-226204.80	-13810247.49	-67.46
	run 1	136.18 [132.64, 139.99]	7.57 [7.43, 7.73]	141.04 [137.97, 144.72]	21.15 [20.81, 21.46]
	run 2	109.42 [105.89, 113.40]	7.03 [6.89, 7.17]	110.43 [106.97, 114.23]	25.75 [25.46, 26.04]
	run 3	128.25 [124.92, 132.32]	7.22 [7.08, 7.36]	126.63 [123.07, 130.51]	28.00 [27.68, 28.36]
	run 4	109.91 [105.89, 114.22]	7.15 [7.02, 7.31]	111.31 [107.07, 115.68]	25.11 [24.76, 25.43]
	run 5	122.03 [118.75, 125.58]	7.36 [7.22, 7.50]	118.19 [114.57, 122.11]	30.06 [29.70, 30.38]
sampl2-**1**	offset	-14157075.01	-202951.46	-13954102.65	-50.95
	run 1	134.57 [131.39, 138.49]	7.42 [7.27, 7.57]	127.84 [124.22, 131.36]	29.36 [29.15, 29.57]
	run 2	118.70 [115.43, 122.45]	8.09 [7.91, 8.27]	110.73 [107.33, 114.69]	29.88 [29.59, 30.17]
	run 3	145.70 [142.59, 149.36]	10.37 [10.13, 10.60]	142.28 [138.90, 145.91]	23.18 [22.70, 23.71]
	run 4	119.81 [115.79, 123.83]	8.38 [8.19, 8.56]	118.88 [115.17, 122.99]	22.54 [22.30, 22.82]
	run 5	132.82 [129.73, 136.32]	8.42 [8.25, 8.59]	132.06 [128.72, 135.87]	22.35 [22.02, 22.67]
sampl2-**2**	offset	-14109609.38	-299336.86	-13810246.31	-59.41
	run 1	121.06 [117.51, 125.02]	9.58 [9.38, 9.79]	106.83 [103.07, 111.19]	37.95 [37.46, 38.42]
	run 2	146.22 [143.00, 150.04]	10.48 [10.27, 10.69]	140.98 [137.87, 144.71]	27.85 [27.47, 28.23]
	run 3	114.17 [110.67, 118.17]	10.27 [10.02, 10.52]	112.73 [109.14, 117.20]	24.47 [24.13, 24.81]
	run 4	141.11 [137.46, 145.20]	10.29 [10.06, 10.52]	136.46 [132.67, 140.67]	27.59 [27.17, 27.99]
	run 5	129.23 [125.77, 133.27]	9.98 [9.77, 10.20]	124.03 [120.72, 127.71]	28.38 [27.93, 28.79]
sampl2-**4**	offset	-14157541.79	-299333.82	-13858178.32	-72.51
	run 1	105.03 [101.24, 108.86]	9.84 [9.63, 10.04]	101.43 [97.69, 105.63]	36.61 [36.33, 36.89]
	run 2	125.33 [121.79, 129.27]	10.20 [9.99, 10.42]	122.30 [118.98, 126.12]	35.66 [35.29, 36.01]
	run 3	104.65 [100.89, 108.96]	9.69 [9.47, 9.89]	97.79 [94.13, 101.69]	40.18 [39.81, 40.57]
	run 4	108.73 [105.16, 112.49]	13.40 [13.17, 13.62]	109.52 [106.11, 113.22]	28.77 [28.18, 29.41]
	run 5	127.61 [124.23, 131.43]	10.07 [9.86, 10.26]	125.32 [122.07, 129.08]	35.24 [34.94, 35.55]

Table S7: Range of minimum and maximum mean potential energies across five runs, reported for the total system ($\Delta\overline{U}^{\text{min,max}}_{\text{total}}$), solute–solute interactions ($\Delta\overline{U}^{\text{min,max}}_{\text{solute-solute}}$), solvent–solvent interactions ($\Delta\overline{U}^{\text{min,max}}_{\text{solvent-solvent}}$), and solute–solvent interactions ($\Delta\overline{U}^{\text{min,max}}_{\text{solute-solvent}}$). Results are shown for MACE-OFF23(S) and ANI-2x. All energy values are given in kcal mol^{-1}.

tautomer pair	$\Delta\overline{U}^{\text{min,max}}_{\text{total}}$ [kcal mol^{-1}]	$\Delta\overline{U}^{\text{min,max}}_{\text{solute-solute}}$ [kcal mol^{-1}]	$\Delta\overline{U}^{\text{min,max}}_{\text{solvent-solvent}}$ [kcal mol^{-1}]	$\Delta\overline{U}^{\text{min,max}}_{\text{solute-solvent}}$ [kcal mol^{-1}]
**MACE-OFF23(S)**				
tp-**558**	6.2	0.2	6.3	0.3
tp-**549**	6.0	0.1	5.6	0.3
tp-**1516**	4.6	3.2	4.7	3.8
sampl2-**1**	3.4	3.0	7.8	7.4
sampl2-**2**	3.2	3.1	3.8	8.3
sampl2-**4**	3.5	2.3	4.8	6.9
**ANI-2x**				
tp-**558**	30.3	1.6	25.9	11.3
tp-**549**	29.5	2.8	30.9	9.5
tp-**1516**	27.0	2.9	31.4	7.5
sampl2-**1**	26.7	0.6	30.7	8.9
sampl2-**2**	31.8	0.9	34.2	13.5
sampl2-**4**	23.0	3.7	27.6	11.4

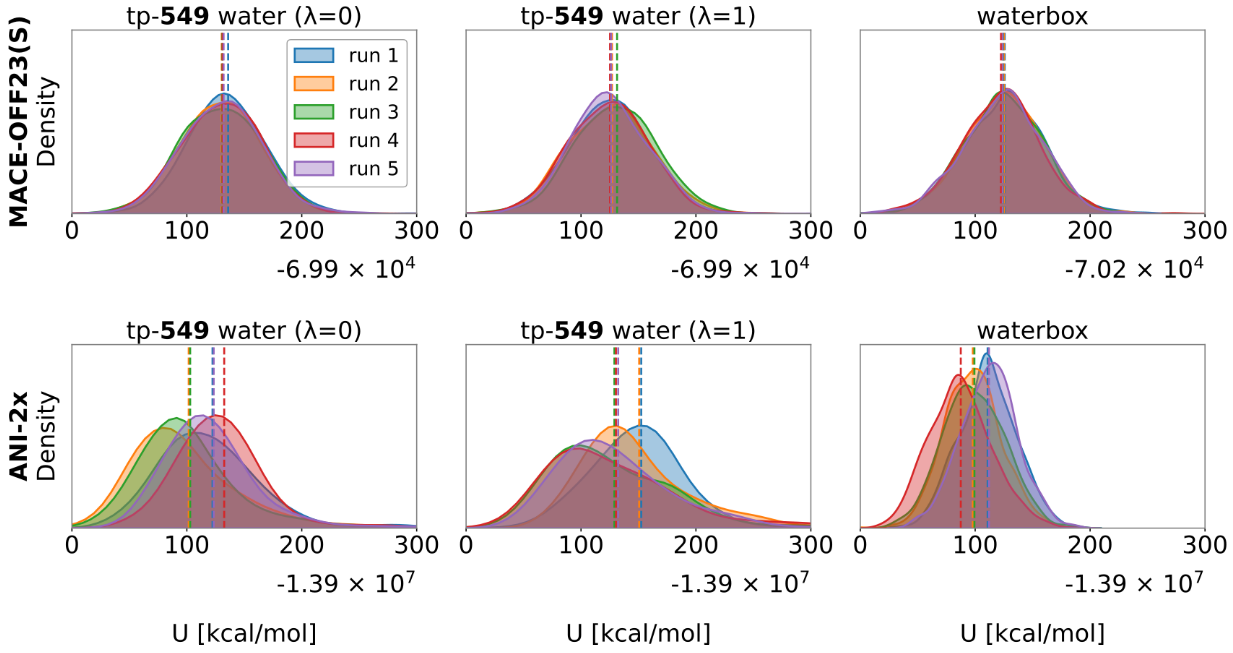

Figure S1: Potential energy distributions of water molecules extracted from tp-**549** solute–solvent simulations at $\lambda = 0$ (first column) and $\lambda = 1$ (second column), compared to pure water box simulations without solute (third column). The water potential energies were obtained by decomposing the total energy of the tp-**549** system into solute, solvent, and solute–solvent contributions. Accordingly, the first column represents the water component of simulations of the tp-**549** enol form ($\lambda = 0$), while the second column corresponds to the water surrounding the tp-**549** keto form ($\lambda = 1$). The first row shows potential energy distributions obtained with the MACE-OFF23(S) model, and the second row with ANI-2x. Potential energies are shown relative to each system's minimum, resulting in a zero offset. Note that the x-axis range is identical for ANI-2x and MACE-OFF23(S). Each simulation was repeated five times, and mean potential energies are indicated by dashed lines.

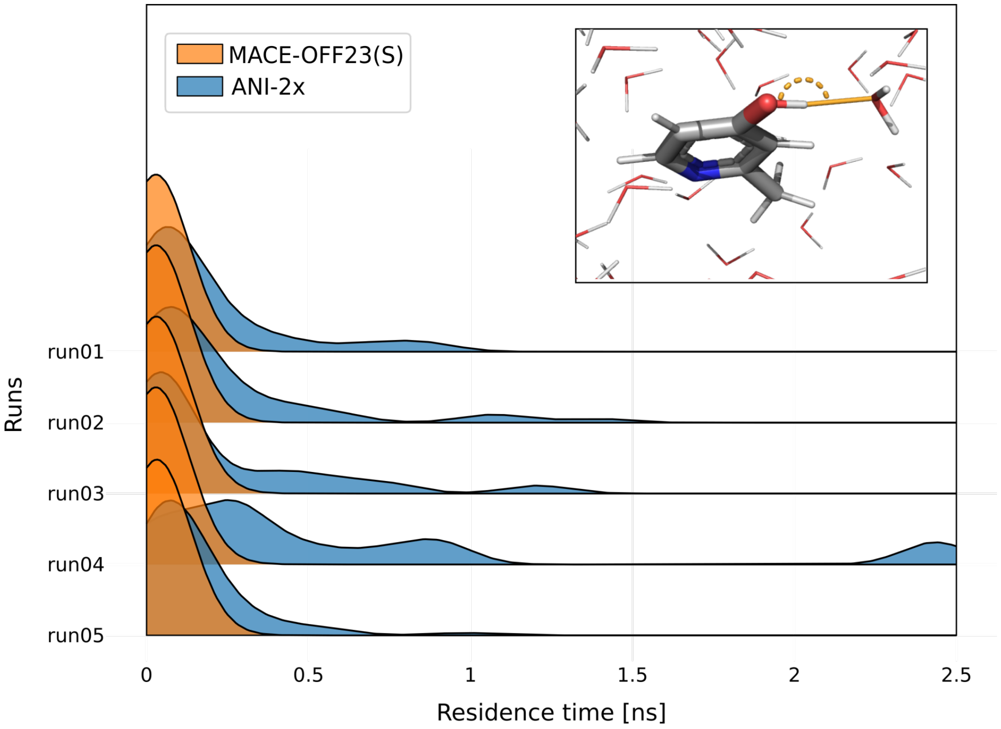

Figure S2: In additional 5 ns simulations, the residence time of water molecules close to the OH group of tp-**558** (enol form) was tracked by applying the following filtering criterion: Water molecules were considered within proximity if their distance was <1.8 Å and the O-H··O angle between the hydroxyl group and the oxygen of a water molecule ranged between 120° and 180° for a given simulation time. ANI-2x results are shown in blue, MACE-OFF23(S) in orange.

References

(1) Taylor, P. J.; Kenny, P. W. The Prediction of Tautomer Preference in Aqueous Solution (Version 1.0). 2019; https://figshare.com/articles/preprint/The_Prediction_of_Tautomer_Preference_in_Aqueous_Solution_Version_1_0_/8966276.

(2) Wahl, O.; Sander, T. Tautobase: An Open Tautomer Database. *Journal of Chemical Information and Modeling* **2020**, *60*, 1085–1089.

(3) Geballe, M. T.; Skillman, A. G.; Nicholls, A.; Guthrie, J. P.; Taylor, P. J. The SAMPL2 blind prediction challenge: Introduction and overview. *Journal of Computer-Aided Molecular Design* **2010**, *24*, 259–279.

(4) Cook, M. J.; Katritzky, A. R.; Linda, P.; Tack, R. D. Aromaticity and tautomerism. Part II. The 4-pyridone, 2-quinolone, and 1-isoquinolone series. *Journal of the Chemical Society, Perkin Transactions 2* **1973**, 1080–1086.

(5) Albert, A.; Phillips, J. N. Ionization constants of heterocyclic substances. Part II. Hydroxy-derivatives of nitrogenous six-membered ring-compounds. *Journal of the Chemical Society* **1956**, 1294–1304.

(6) Evans, D. A.; Smith, G. F.; Wahid, M. A. The tautomerism of 3-hydroxyisoquinolines. *Journal of the Chemical Society B* **1967**, 590–595.
